# Advancing vascularized composite allotransplantation: essential factors for upper extremity transplant program development

**DOI:** 10.3389/frtra.2024.1406626

**Published:** 2024-06-11

**Authors:** Martin Kumnig, Caroline Kobler, Alessandra Zaccardelli, Gerald Brandacher, Simon G. Talbot

**Affiliations:** ^1^ Department of Psychiatry, Psychotherapy, Psychosomatics and Medical Psychology, Center for Advanced Psychology in Plastic and Transplant Surgery, Medical University of Innsbruck, Innsbruck, Austria; ^2^ Division of Plastic Surgery, Brigham and Women’s Hospital, Boston, MA, United States; ^3^ Department of Visceral, Transplant and Thoracic Surgery, Center of Operative Medicine, Medical University of Innsbruck, Innsbruck, Austria; ^4^ Department of Plastic and Reconstructive Surgery, Vascularized Composite Allotransplantation (VCA) Laboratory, Johns Hopkins University School of Medicine, Baltimore, MD, United States

**Keywords:** vascularized composite allotransplantation, bioethical issues, program development, upper extremity transplantation, patient selection, rehabilitation, multidisciplinary collaboration, qualitative research

## Abstract

Vascularized Composite Allotransplantation (VCA) offers a unique option to restore form and function after limb loss or facial trauma that cannot be satisfactorily accomplished through traditional prosthetics or reconstructions. Establishing a successful Upper Extremity Transplantation (UET) program requires strong leadership and a structured surgical team, and extensive interdisciplinary collaboration. We conducted a qualitative study among 12 health care professionals and patients. Informed consent was obtained per protocol, and semi-structured interviews were conducted online and recorded. Participants reported their perceptions of factors that either favored or hindered a successful outcome, including functional status before and after surgery, preparation for transplant, shared decision-making, rehabilitation, and psychosocial support. Thematic analysis revealed that it is essential to establish a team comprising various disciplines well before performing VCA procedures. Defining a common goal and choosing a defined leader is a key factor in procedural success and requires open collaboration beyond what is typical. Primary described categories are interdisciplinary collaboration and skills of the VCA team, building and leading a VCA team, pre-transplant procedures, post-transplant course, and factors to consider when establishing a program. The basic roles of team science play an outsized role in establishing a VCA program. Transplantation medicine involves various overlapping scientific and medical categories requiring health professionals to consciously work together to establish complex vertical and horizontal communication webs between teams to obtain positive outcomes. In addition to medical considerations, patient-specific factors such as transparent communication, therapy plan establishment, plan adherence, and continual follow-up are significant factors to overall success.

## Introduction

Vascularized Composite Allotransplantation (VCA) has emerged as a promising reconstructive technique to restore form and function among patients suffering from devastating limb and facial injuries. Given the complexities intrinsic to composite grafts, implementation of an upper extremity (UE) VCA program requires meticulous planning, robust infrastructure, and multidisciplinary collaboration. In this manuscript, we will explore these crucial elements, ranging from surgical expertise and immunosuppression protocols to ethical considerations and long-term sustainability. By illuminating the often-unseen intricate tapestry that forms the foundation of a thriving VCA initiative, we hope to highlight overlooked criteria that will help guide medical institutions and practitioners toward establishing and fostering successful VCA programs, ultimately benefiting patients worldwide.

VCA involves melding multiple intricate microsurgical techniques, requiring the expertise of microvascular, orthopedic, and plastic surgeons working together to repair various tissue types, while concurrently efficiently communicating to reduce ischemic time (1–3). Meanwhile, effective donor procurement is integral to procedural success and necessitates a strong relationship with local organ procurement organizations (OPOs). This may be achieved via frequent meetings with OPOs and practice procurements (4–6). The procurement process must optimize donor identification as well as donor-recipient matching for immunological compatibility to minimize the substantial risk of rejection. This in turn requires expertise in transplant immunology to establish immunosuppression protocols determining appropriate patient-specific regimens to prevent rejection while mitigating side effects and complication risks. Overarching the above considerations is the perioperative period, which requires a complex balance of physical and psychological support (7–12). UE transplantation is both physically and psychologically taxing, limiting its suitability to a subset of the patient population who are both motivated and prepared for the inevitable challenges and setbacks during recovery. This is best accomplished via rigorous patient selection criteria and comprehensive evaluation of potential recipients including medical/physical, psychological, and social support factors to ensure they can cope with the procedure, and adhere to the post-transplant care plan (7, 13). An interdisciplinary team of psychologists, physical therapists, and occupational therapists should be involved in the pre- and post-transplant rehabilitation process as recipients prepare for surgery, then work to regain functional use of the transplanted limb (14). For instance, psychiatric teams should assess patients’ ability to accept the new graft as part of their body, as well as coping mechanisms they possesses that will help them navigate media attention, ongoing intensive medical care, and intimate reliance on caregivers (12). Specifically, psychiatric providers must screen for underlying psychiatric conditions, perceptions of body image, overall quality of life, and ability to adhere to follow-up care (8). Concurrently, other team members must establish the complicated rehabilitation protocols required for successful VCA, which include elements such as preoperative functional assessments, initial postoperative evaluation, intermediate therapy, and late maximization of function (15). Thus, a successful VCA program requires not only exceptional surgical skill, but open and detailed collaboration among various medical groups, including transplant medicine, immunology, rehabilitation, psychiatry, and social work.

Outside of the procedure itself, there are multiple domains to consider. For instance, VCA raises a host of other issues, such as informed consent, donor source, and allocation policies (16, 17). Ensuring compliance with these ethical, legal, and regulatory requirements is essential to the establishment and functioning of a VCA program**.** At the same time, VCA is a costly procedure, and securing adequate financial support and resources for the program's sustainability is crucial. This includes funding for surgeries, immunosuppressant medication, follow-up care, and research. In particular, a commitment to ongoing research and innovation is essential to improve outcomes, reduce complications, optimizing the procedure's cost/benefit ratio, and thereby improve future odds for long-term success. Similarly**,** raising public awareness about VCA can help foster support for the program both financially and by increasing the number of potential donors.

Overall, successful development of a VCA program for upper extremities requires a dedicated and multidisciplinary approach that considers medical, ethical, financial, and logistical aspects to provide the best possible outcomes for patients.

## Materials and methods

### Participants

All participants in the study were either patients that underwent UE VCA, recipients’ relatives, or health care professionals in the interdisciplinary VCA team at the Medical University Hospital Innsbruck. Participants received an introductory email requesting participation in an online interview and provided written informed consent. All study activities were approved by the Ethics Committee of the Medical University Hospital, Innsbruck (vote-nr. 1036/2023).

### Interviews

Interviews were conducted between 04/2021 and 08/2021. We conducted a total of 12 interviews: four with UE VCA transplanted patients, one with a patient's relative, and seven with UE VCA health care professionals. A trained interviewer conducted the interviews using a semi-structured guide. The interview guide consisted of open-ended questions that asked participants about their perceptions of factors that either favored or hindered a successful transplant outcome. Topics included functional status before surgery, experience with the preparation for transplant, decision process and information transfer, rehabilitation and functional outcome after surgery, and family and social support. Interviews were conducted online and recorded with the interviewees’ consent. Subsequently, a qualitative analysis of the interview transcripts was performed.

### Thematic analysis

**Figure 1:**
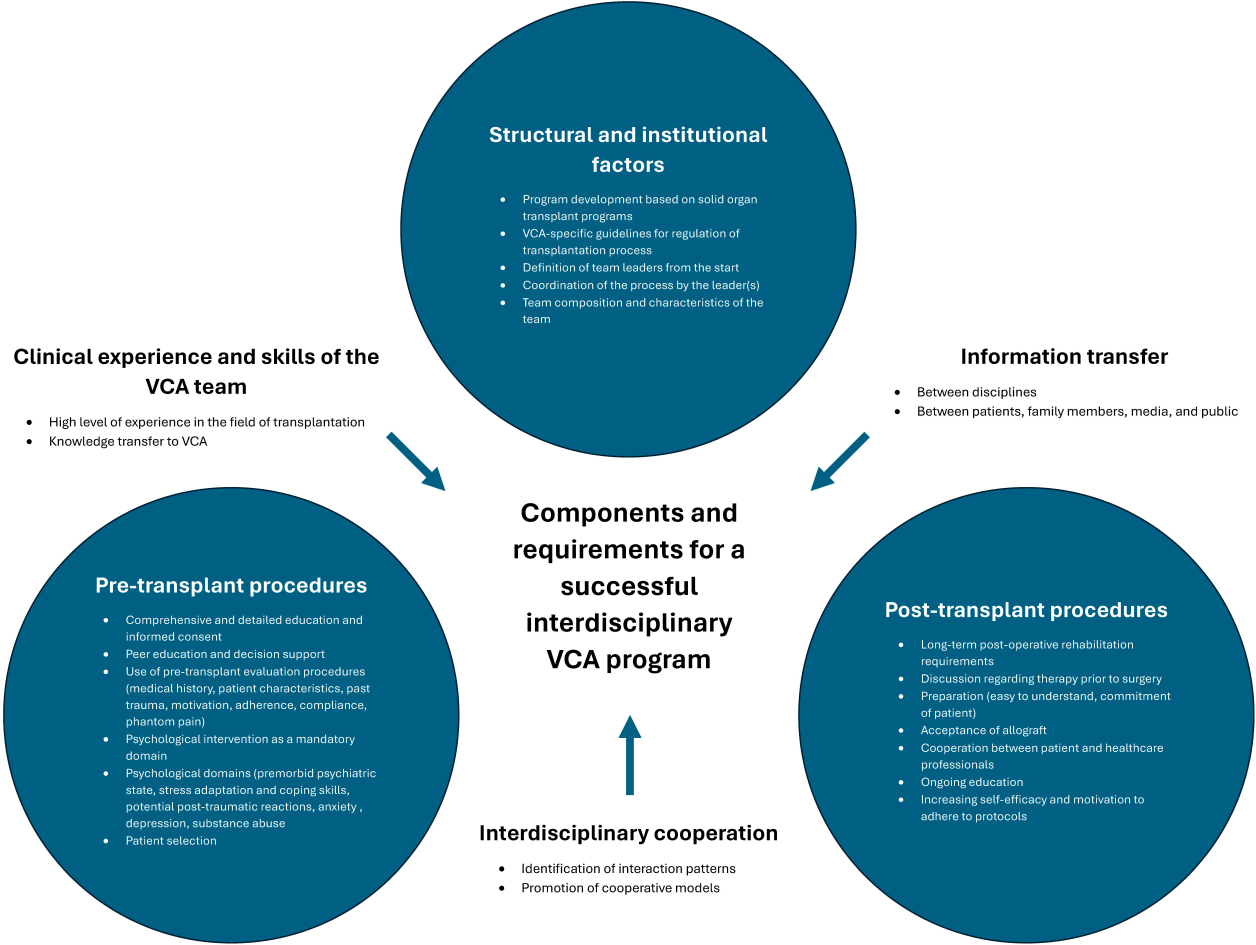
Thematic map..

Thematic analysis was performed utilizing a standard qualitative methods. Salient words or phrases pertaining to factors influencing UE VCA success were coded into 51 codes and 10 subcodes which were applied to all transcript data. Codes were then categorized into themes, ensuring appropriate internal homogeneity shared within groups but with sufficient external heterogeneity to be distinct from other themes. We used qualitative analysis software (Atlas.ti, https://atlasti.com/) to extract citations supporting the themes. The interview recordings were reviewed to identify themes, using the qualitative analysis software to index their digital location in the recordings. All investigators agreed upon a thematic map showing relationships between the individual themes and the guiding questions of the study (see Table 1 and Figure 1).

**Table 1:** Categories and themes from the thematic map with samples of interview transcription.

Categories	Themes	Sample of supporting text from transcripts
Interdisciplinary cooperation and skills of VCA team		
Clinical experiences and skills of the interdisciplinary VCA team	High level of clinical experience in the field of transplantation medicine and surgical as well as other skills of the interdisciplinary team are important to transfer this knowledge to VCA.	“A profound knowledge in solid organ transplantation is very important. “.. (transplant surgeon TS) “It should be done at a transplant center or with a transplant center. “…. (TS) “A good basic knowledge as regards general transplantation.” (TS)
Cooperation between disciplines and interactions within the VCA team	Identification of interaction patterns between disciplines and promotion of cooperative models that support the recognition of an interdisciplinary VCA team.	“If you see the whole picture you have to define a goal already in the RTI. A patient, an age, a motivation… Then you can set a goal that you are able to achieve.” (plastic surgeon PS)
Information transfer between disciplines	Definition of modalities that define the information transfer between disciplines/within the team, patients and family members. Providing tools that support a fast information transfer that avoids linguistic barriers on a low threshold basis.	“With these patients, you have to recognize when you need to consult another discipline. You have to ask people who have the same clientele if they have seen this or that before. You have to inform yourself.” (TS)
Building up a VCA team and leading it		
Leadership	Because of the complexity of communicating and interacting in an interdisciplinary team, there has to be a leader of the VCA group. This hasn’t to be a team members but a highly experienced person in the field of VCA that coordinates and moderates all processes within the group.	“such centers must have a good team spirit and a clear goal” (PS) “For this to work well, there needs to be a strong lead and also the willingness of the individuals to participate.” (PS)
“Becoming a team and working as a VCA team”- Teamwork	Elimination of hierarchical thresholds, welcoming communication attitudes, all actions are based on a team effort.	“In my team there is a flat hierarchy, but still one decision-maker. There are no solo efforts including the boss, everything is discussed in the team what is useful and what is not.” (hand surgeon HS)
Team composition	The VCA program is a joint interdisciplinary work and therefore the team must be built well-considered. Each discipline represents a pillar for success and is equally as important as the other.	“This is a joint interdisciplinary work, where all pillars are equally important. And so we have to respect each other and see which way is best to organize everything together.” (functional therapist FT)
Pre- transplant procedures: decision-making, education, examinations		
Education, informed consent and decision making	Comprehensive and detailed education and informed consent process during the whole VCA procedure (pre- as well as post-transplant). Support of patients in their decision-making process through comprehensive and detailed information as well as close contact with the physicians, but avoid making the decisions for him.	“Very important to guide a patient to a decision and help, but to avoid making the decision for someone. So that the result can be satisfactory.” (TS) “I think what is very important to find out what exactly is the reason why the patient would like to have this. And to what extent he has already dealt with alternatives, whether there were problems. Whether there were identification problems.” (TS)
Peer education	Support of the information exchange between already transplanted patients and VCA candidates through the whole process. Completing the candidates’ recognition of VCA by sharing experiences on graft function, quality of life improvement, etc. but also on potential negative experience/effects of VCA procedures.	“I think that this is the key for such an orientation meeting or exploratory meeting and that everything else would be initiated from this point. I think that should be the beginning.” (TS) “That would be very interesting for the patient when he sees that (the already transplanted) then. And then maybe also sees what they can do with their hands and what their hands look like. I think that is the most important thing for peer education.” (TS)
Medical history and patients’ characteristics	Assessment of the preoperative history and medical background is also meaningful for the decision making process and potential post-transplant outcome, particularly the identification of patients’ past trauma, motivation level, adherence, compliance and phantom pain.	“It is very important that the trauma is at least to a large extent overcome… ..that the patients are stabilized and can cope with everything else that comes with a strong psyche.” (PS)
Pre-transplant evaluation procedures (in general)	The successful outcome of transplantations requires the careful selection of transplant recipients trough different medical screenings and evaluation. Development of a standardized protocol that defines the pre-transplant evaluation, including all relevant procedures for potential VCA surgery, based on institutional and national standards as well as scientific recommendations. The pre-transplant medical history should include a full evaluation of the patients past, present, and future risk of exposure to infection. The medical history also includes, e.g., patient´s current medical problems including active infections, prior infections, allergies, intolerances to medications and the complete vaccination history. Prior the transplantation medication allergies should also be reviewed critically to assess the nature and severity of prior drug reactions.	“We then also said that we would like to have a score where you go through everything psychosomatically and psychologically and then say: this one would be the absolute candidate and this one rather not.” (PS) “The clarification is very standardized, it works very well. (TS) It is important to examine the patient in hospital… … that you know exactly: on the basis of these examinations, I can plan and assess. The transplant clarification, immunological, exclusion of infection, exclusion of malignancy is a standardized procedure that works perfectly.” (TS)
Pre- transplant psychological evaluation	One mail goal of the evaluation is to select patients that most likely benefit from the VCA transplantation. Also the identification of areas of psychological intervention, before and after transplantation, is mandatory. The psychological evaluation is part of the standardized pre-transplant protocol that has to assess main psychosocial domains: patient´s premorbid psychiatric state, their adaption to various stressors and coping skills, history of medical treatment and adherence. The evaluation should also include potential posttraumatic reactions, anxiety, depression as well as substance abuse history, health-related quality of life and general health behaviors.	“A psychological evaluation of the objectives, the motives as well. That would also be important.” (psychiatrist psych) “Psychologically, the patient must be completely screened. There must be no doubt about the postoperative rehabilitation. That he is then satisfied with the result.” (PS)
Post transplant course and factors		
Post-transplant rehabilitation	Long-term post-operative rehabilitation is needed to provide good results after VCA transplantation daily training, intensive collaboration between disciplines and patients, strong patients’ adherence to the post-transplant treatment protocols. Satisfying functionality of the allograft can only be provided by commitment with rehabilitation procedures and profound relationship between rehab professionals and their patients. Physiotherapy and rehabilitation protocols should be discussed and planned before transplantation so that they can then be started immediately after VCA. The definition of common goals in between disciplines is important. Precise work as well as accurate patient education is of great importance in the field of immunosuppression to avoid any kind of rejection reactions.	“Intrinsic motivation as one of the main factors for the successful rehabilitation.” (psych) “For the patient himself, a psychological preparation for what is to come is quite important.” (PS)
Factors that contribute to VCA failure	Like a successful VCA transplant is based on the team performance, unsuccessful transplantation can be a result of both, patients’, and doctors’ failure. A very important factor for a successful transplantation is the patient's acceptance of the allograft. The patient's inner attitude plays a central role, especially the body's acceptance of the new transplant. According to health care professionals, self-discipline, a structured daily routine and a clear idea of how to use the “new” hands are productive factors.	“Someone who has no regular daily routine, someone who has no idea what he would like to do or could do in the future. So someone who basically hangs in the air with everything.” (TS) “The unsuccessful rehabilitation mainly makes the difference in the head. These are my new hands, and they are beautiful…. This is a successful transplant. An unsuccessful one is just the opposite. If I don't see these hands as my new hands.” (patient pat)
Factors that contribute to successful outcomes after VCA	There are several different factors that contribute to the desired outcome, but it is not possible to say that these factors directly influence it. A structured plan with a close contact person as well as a good team composition, especially a strong leader and the willingness of the others to follow his ideas and instructions are part of these factors.	“The fact that a contact person and also reference persons are always present, that is especially important.” (HS) “It's like a kind of marriage that you enter. You are in regular contact and have a very close relationship, and this exchange is very important.” (psych)
Patients’ characteristics that contribute to success	The patient's motivations to adhere to pre- and post-transplant protocols is very important. To achieve the best outcome, it is therefore of great importance to ensure this adherence. It starts already before the surgery through comprehensive information about possible effects, side effects and complications. Patients also report that close contact with physicians plays a central role and they are then more likely to report problems, e.g., early rejection reactions. Another factor not to be neglected for a successful transplantation is that the patient himself is convinced of the transplantation and wants it with full conviction. The more convinced the patient is, the more likely it is that the transplant will be successful.	“The patient must establish a partnership with the doctor who treats him.” (TS) “The transplant patients must contact us quickly as soon as something does not seem right. The doctor must be available. A patient like that is a big challenge and it requires a team to handle it. It also takes a lot out of the medical staff. “ (TS) “You have to create this relationship of trust that they are supposed to simply get in touch.” (TS)
Team composition and professionals’ characteristics that contribute to success	Team structure as well as possible leaders should already be determined before the transplantation, so that then in the entire process as few problems and questions as possible arise. Good preparations before, during and after the transplantation is also important. Structured plans for the physicians but also good therapy plans for the patients play a central role. Therapy plans must be easy-to-understand to ensure patient´s compliance as best as possible.	“That was good, this thinking and working together. Everyone at the same level and with respect. And that also contributes to success.” (PS)
Establishing up a VCA Program		
Program development	A VCA program needs to be developed on the basis of a successful solid organ transplant program, focusing on an interdisciplinary team effort, merging the knowledge and skills of all disciplines that are part of this team. Ongoing program development based on the team experiences and scientific insights.	“…to have an entire program already from the start” (PS)
Creating guidelines	Based on the commitment of the interdisciplinary team members on treatment standards, specific VCA guidelines should be developed that guide the different pre- and post-transplant procedures. Guideline development can start on the protocols already developed in the field of solid organ transplantation, including all relevant factors that are unique for VCA transplantations.	“Of course, these are all individual decisions, but you should still follow a standardized protocol.” (PS)

Health care professionals (shortcuts): PS, plastic surgeon; TS, transplant surgeon; HS, hand surgeon; FT, functional therapist; Psych, mental health care professional; Pat, patient; Rel, relatives.

## Results

Patients, their relatives, and healthcare workers identified five critical components of a successful VCA program: interdisciplinary collaboration and VCA team skills, building and leading a VCA team, pre-transplant procedures, post-transplant course and factors, and establishing a VCA program. There is a dynamic interplay between these factors; for instance, efficient interdisciplinary collaboration and the development of team skills influences the ability of the team to build and lead, which in turn influences the implementation of thorough pre-transplant procedures and the understanding of the post-transplant course. It is the interaction of these components both among themselves (i.e., horizontally) and between one another (i.e., vertically) that underlines the holistic nature of building and sustaining a successful VCA program.

### Interdisciplinary cooperation and skills of VCA team

Participants emphasized the importance of clinical knowledge, stating “…a profound knowledge in solid organ transplant is very important.” A significant level of clinical expertise in transplantation medicine, coupled with reconstructive surgical proficiency and a diverse skill set within the interdisciplinary team, is crucial for effectively applying this knowledge to VCA.

This involves pinpointing interaction patterns between different disciplines and fostering collaborative models that enhance the cohesion of an interdisciplinary VCA team. One respondent stated, “With these patients, you have to recognize when you need to consult another discipline.” Challenges to collaboration during procedures may include unrecognized biases by doctors against crossing surgical subspecialty silos, lack of familiarity with OR and support staff protocols by team members who may not often collaborate given the relative rarity of VCA procedures, and unspoken assumptions regarding the responsibilities and skill sets of participants. It also involves establishing clear modalities for information exchange beyond surgeons to other team members, patients, and their families. Equipping the team with tools that facilitate swift information transfer, while minimizing language barriers on an accessible platform, is essential. In this domain, challenges range from time pressures on team members and coordination of responsibilities for disseminating complex information to the potential for misunderstandings by patients and their support groups due to lack of context and the volume of medical information to be digested. Thus, equipping teams with tools that facilitate swift information transfer while minimizing vocabulary and language barriers is essential.

### Building a VCA team and leading it

As discussed above, VCA programs extend far beyond the OR. The comprehensive care team encompasses experts and their support staff in Plastic Surgery, Orthopedic Surgery, Transplant Surgery, Transplant Medicine, Radiology, Pathology, Dermatology, Occupational and Physical Therapy, Psychology, Social Work, and Ethics (4). Given the complexity of these teams and the specialized knowledge they possess, selection of an experienced leader with team management skills is imperative to ensure group cohesion. This is not always the most senior team member. While their gravitas may be an asset, more important is the ability to collaborate in ways that encourage speaking freely, and challenging experts in diverse fields to consider other team members’ opinions. Once established, the leader must take responsibility to actively mediate group deliberations and ensure expertise from each discipline is first collected horizontally from all levels within a team, and then properly evaluated and disseminated vertically between teams. This sentiment backed repeatedly in interview responses stating for example “(F)or this to work well, there needs to be a strong lead and also the willingness of the individuals to participate.” In this way, VCA participants emphasize the importance to them of group deliberation and the intentional inclusion of all team members by a leader capable of executing this difficult assignment. For example, one participant stated, “In my team there is a flat hierarchy, but still one decision-maker. There are no solo efforts including the boss, everything is discussed in the team what is useful and what is not.”

### Pre-transplant procedures: decision making, education, and examinations

Prior to initiating treatment, clinicians must engage in shared decision-making with VCA candidates. Patient-centered language should be used to discuss each aspect of the procedure including preoperative screening, pharmacological therapies, recovery and rehabilitation, potential complications, and alternative options. Interviewees highlight the careful balance clinicians must take, stating it is “… very important to guide a patient to a decision and help, but to avoid making the decision for someone, so that the result can be satisfactory.” Clinicians in all disciplines should be pressed to acknowledge and emphasize patient autonomy as the ultimate arbiter in choosing whether to proceed with treatment, something that can be downplayed by even the most well-meaning practitioners.

Although easy to overlook, VCA patients also emphasize the benefits of exchanging information with other recipients. In particular, witnessing grafts first-hand helps prepare patients viscerally for their own choice, while sharing experiences affords them a sense of community. Thus, early integration with other VCA patients should, if practicable, be considered a mandatory part of patient education. Additionally, the foundation of VCA patient support groups is very important, e.g., the recently initialized international group “VCA world” (url: www.worldofvca.com).

In addition, potential VCA recipients must undergo extensive preoperative evaluation. Initially, assessments of patients’ past medical histories can be used to evaluate potential candidates. Patients must then undergo extensive additional screening prior to be listed for allotransplantation including preoperative testing with transplant medicine, renal, infectious disease, critical care, and rehabilitation services. Other workup includes routine transplantation laboratory testing, electromyography/nerve conduction studies, and imaging of both upper extremities.

Meanwhile, a comprehensive psychological evaluation must be an integral component of the standardized pre-transplant protocol. Patients undergo a thorough psychiatric history including post-traumatic reactions, anxiety, depression, and substance use. Clinicians must also ascertain their coping mechanisms and ability to adapt to different stressors as well as their level of motivation and adherence to previous medical treatments and their present level of motivation. Assessing health-related quality of life and general health behaviors is also essential.

### Post-transplant course and factors

Achieving optimal results after a hand transplantation requires extensive post-operative rehabilitation. Daily training, close collaboration between various disciplines and patients, and strict adherence to treatment plans are pivotal for success. Intrinsic motivation stands out as a key factor in driving successful rehabilitation, and by extension, transplantation. Patients must undergo psychological preparation to ready themselves for what lies ahead. The team must also find out what is driving a patient's desire for VCA, as stated, “I think what is very important to find out what exactly is the reason why would like to have this. And to what extent he has already dealt with alternatives, whether there were problems.”

Physiotherapy protocols should be thoroughly discussed in an interdisciplinary manner and planned well in advance of the transplantation to avoid any delays in initiating the rehabilitation process. Disciplines must work vertically in a cohesive and open manner to establish mutual objectives. Then, educating patients about their responsibilities in the success of the VCA in language they can internalize is of paramount importance, particularly regarding immunosuppression. As a life-long and largely self-administered immunosuppression plan is required to prevent rejection, safeguarding the success of the procedure and the recipient's well-being cannot be achieved without full patient participation.

Certain characteristic patterns contribute significantly to success. As mentioned, the patient's motivation and adherence are crucial. As stated below, “Intrinsic motivation as one of the main factors for the successful rehabilitation.” Patients need to trust those responsible for their care, which is built step-by-step from the initial conversation and then throughout the VCA process via techniques including transparent communication delivered without hyperbole. Caregivers should acknowledge the primacy of patients’ viewpoints by showing empathy toward their emotional and physical challenges, and by encouraging them to participate in decision-making about their care, providing agency and enhancing trust. Doctors should be prepared to answer queries repeatedly, and contact persons, including physicians, surgeons, and medical staff, should be as accessible as possible as a close relationship is imperative for reporting any issues. Dr. Kumnig uses the phrase, “It's like a kind of marriage you enter …”

The patient's inner attitude is closely linked to the success of the transplantation as acceptance of the new transplant is pivotal. Patients must integrate the new allograft as part of their own body. They need to feel it, see it, and use it. In the interviews, the patients themselves expressed that they perceived the new hand as an integral part of their own body, emphasizing they felt this perception was instrumental in achieving significant success.

In summary, the composition and attributes of the professional team hold significant importance in this context. Potential leaders should be identified prior to transplantation. The planning stages—before, during, and after the surgery—are essential and must be precisely executed. Collaboration should be rooted in respect, with disciplines functioning as one cohesive team. Therapy plans should be straightforward to ensure patient adherence to treatment plans.

### Establishing a VCA program

To ensure the success of a transplant, it is imperative to have a comprehensive VCA program in place from the outset. These programs should be built on the foundation of established programs for solid organ transplant, with a primary emphasis on interdisciplinary teamwork. The program should draw from the collective experiences of the team and be guided by current scientific knowledge and insights.

Adherence to standardized protocols and guidelines is essential. Specific guidelines tailored for VCA must be established to provide a clear framework for professionals to navigate through pre- and post-transplant procedures. The development of these guidelines should start early on and draw from the field of solid organ transplantation, while also incorporating factors that are unique to VCA. On respondent stated, “Of course, these are all individual decisions, but you should still follow a standardized protocol Of course, these are all individual decisions, but you should still follow a standardized protocol.”

While individual decisions may be necessary for each VCA transplant, it is crucial to adhere to standardized protocols to ensure consistency and optimal outcomes.

## Discussion

We have strived to illuminate patient, family, and healthcare worker perspectives, values, and expectations of VCA programs. Our data provides valuable insights into the areas where providers’ and patients’ opinions are in agreement, and where they differ. Notably, while providers frequently commented on the structural elements of VCA programs, patients, perhaps unsurprisingly, more often discussed the psychosocial impact of the procedure. Combining these at times disparate viewpoints yields a more robust understanding of the critical components of a VCA program. Through this lens, we identified central themes including interdisciplinary cooperation and skills of the VCA team, forming a VCA team and leading it, pre-transplant procedures including decision-making, education, examinations, post-transplant course and factors, and establishment of a viable VCA program.

These themes are of particular importance when establishing a UE program given the unique aspects of VCA compared to the more extensively studied solid organ transplantation (SOT). In particular, VCA is a non-lifesaving treatment requiring substantial ongoing medical, psychological, and social support. While SOT recipients typically rely on their transplanted organ for survival, making transplantation more urgent, VCA patients are generally medically stable. Thus, providers have the luxury to spend more time choosing appropriate candidates and conducting screening procedures while patients have the leeway to deliberate if the procedure and team are a good match for their goals. In addition, VCA is a very complex surgical procedure, and rehabilitation and clinical outcomes are highly dependent on patient participation, necessitating identification of motivated, psychologically stable candidates with ample psychosocial support systems. Limb transplantation itself leads to substantial patient stress related to lifestyle changes, media attention, and inevitable reliance on family and friends, further underscoring the importance of a robust support system. At the same time, patient and family support, education, and advocacy is critical in discussing immunosuppression, the experimental nature of some aspects of these operations, and plans for failure including explantation to ensure patients are making a fully informed decision when consenting to undergo this elective procedure. Finally, VCA involves complex teamwork and requires strong leadership, collaboration between specialties, and appropriate hospital infrastructure.

Ethical and legal considerations vary between both transplantation programs, as they are influenced by the nature of the transplanted tissues and their impact on identity and autonomy. These issues may diverge from those encountered in SOT. In comparison to functional outcomes and quality of life, VCA has the potential to restore complex functions such as motor control, sensation, facial expressions what then significantly improves the quality of life. It's imperative that these aspects are thoroughly discussed as part of informed consent procedures and carefully considered in treatment plans. SOT prolong life and improve health, it's important to note here that it not always restores the full range of organ function or address aesthetic concerns.

It's evident that there are differences between these two organ transplantation procedures, highlighting the necessity for specialists and tailored approaches in both. Additionally, it's crucial to emphasize that both types of transplantations require an interdisciplinary approach and collaboration with both medical and non-medical professionals.

VCA relies on a near-seamless integration of expertise from various specialists, whose scope of practice may not otherwise overlap, in order to effectively complete an infrequent and challenging procedure. In this domain, team science can help explain how to promote communication and collaboration to achieve the common goal of optimized patient outcomes. Griffith et al. investigated the role of team dynamics in VCA programs, finding that regular ongoing meetings such as potential recipient reviews and discussions of transplantation criteria—even in the absence of an active candidate—helped to foster team cohesion and the contribution of individual expertise. In addition, practice sessions to refine protocols, rehearse transplantation techniques, and review safety and transitions improved procedures and helped identify potential areas requiring additional planning and training (18, 19). In cases of unexpected complications or emergencies, a well-coordinated team is essential for making rapid decisions and providing the best possible care (18, 20–22).

VCA also requires substantial lifelong commitment from the recipient. In selecting VCA candidates, clinicians must help patients carefully weigh the benefits (i.e., return of function and potentially improved quality of life) with the substantial risks (i.e., graft rejection and overall decreased life expectancy) while concurrently emphasizing patient autonomy. Dumont et al. proposed strategies to facilitate informed decision-making including absence of urgency to decide, acceptance of uncertainty of outcomes, and space for reluctance as the patient grapples with the possibility of transplantation (23). Novel avenues to thoroughly communicate with patients regarding the VCA process have been proposed; notably an eLearning program has been designed to train donor professionals which yielded improved clinician confidence and may help improve provider-patient communication (24). Similarly, educational websites have been developed to provide patients with educational information and found to have high patient satisfaction (17).

Patients opting for allotransplantation must then undergo a substantial medical and psychological workup. Though no universal selection guidelines have been established, a systemic review analyzing shared criteria between various facial transplant programs determined that common factors include e. g. psychological stability, overall appropriate physical status, and medical adherence (25). Meanwhile, Laspro et al. found that eligibility criteria for hand transplantation were consistently centered around capacity to manage the allograft, including access to follow-up, insurance coverage, psychological stability, history of medical adherence, but noted that factors related to immunosuppression were less emphasized (26). From a patient perspective, the most important themes regarding UE VCA selection criteria are younger age, good physical health, mental stability, willingness to “put in the work,” presence of specific amputation characteristics, and sufficient social support (16). Understanding such perspectives aids clinicians in aligning with patient expectations of the allotransplantation experience, and leads to enhanced patient education regarding the process (24).

Indeed, identification of an enthusiastic, motivated, and psychologically stable candidate equipped to cope with unique physical and psychosocial challenges is paramount (9). Psychological assessment procedures including the diagnostic interview as well as instruments such as the Psychosocial Assessment of Candidates for Transplantation (27) and the Stanford Integrated Psychosocial Assessment for Transplantation (28) have been employed. In addition, it is important to assess patients’ psychiatric history, body image adaptation to trauma, cognitive preparedness, coping techniques, and motivation (10). Kumnig et al. determined that, among hand transplant candidates, psychological impairments commonly include social withdrawal, embarrassment, and poor quality of life; while motivation for transplant varied based on bi- versus unilateral impairment, native versus accidental hand loss, and degree of social integration (11). In addition, lower-risk candidates have been identified as those with realistic expectations and ability to actively participate in physical therapy (13). Of note, psychosocial factors have been demonstrated to be predictive of clinical outcomes among solid organ transplant recipients—a phenomenon which may extend to VCA patients (29). In particular, depression and impaired cognitive function, and posttransplant psychological factors are associated with increased clinical risk among SOT patients. Meanwhile, psychological flexibility and self-efficacy may be protective (29). In VCA UE recipients, rejection has been observed in 33% of patients with depression, 22% of those with posttraumatic stress disorder, and 17% of individuals with anxiety (7). These factors may contribute to patients’ motivation to adhere to the lifelong therapies required to sustain the allograft. Several studies have indicated a higher incidence of organ-related complications and an elevated risk of organ rejection in non-adherent patients. Unsurprisingly, the need for expert interdisciplinary collaboration permeates into the psychosocial domain as well. Hummel et al. conducted semi-structured interviews among UE staff, transplant patients, and relatives and determined that interdisciplinary teams with adequate resources for patient selection as well as ongoing close provider involvement contribute to transplantation success (30).

Similarly, social work departments play a vital role in ensuring that a robust social support system is in place as patients work to regain a sense of self, body image, and identity (9, 12, 30–32). In particular, patients and their caregivers have identified community and caregiver support as crucial factors in VCA success (33, 34). Regrettably, inclusion of social criteria in candidate selection introduces an inherent inequity; and as VCA becomes more mainstream it will be incumbent on researchers to determine the association between social support and patient outcomes, and how to attenuate social risk among marginalized individuals with fewer resources.

Achieving a successful transplant outcome requires the active involvement and dedication of the patient. Adherence to medical advice, open communication with the transplant team, and utilization of social support networks are pivotal elements in ensuring long-term transplant success.

### Limitations

There are several limitations of this work including the small sample size of 12 participants. This reflects an intrinsic challenge of VCA research as the patient population worldwide is quite limited, and thus better suited for qualitative, rather than quantitative, analyses. Another limitation is that our data is skewed by the larger proportion of interview responses from healthcare providers than patients, making the sentiments of VCA team members more heavily represented. Thus, our results primarily focus on provider opinions with complementary insights from VCA recipients. In addition, we identified discrepancies in the content of responses between providers and patients. This is likely related to an additional question about the psychological impact of VCA which was included in the patient interview, prompting VCA recipients to focus on their individual experiences while providers discussed the more structural elements of the program. In a related fashion, patients, who are more intimately familiar with their own VCA journey are more likely to comment on this aspect of the process than providers. Another limitation is the absence of media-related aspects in the interview. There was no discussion about how the public media reacted to transplant recipients and how extensively information about allotransplantation is disseminated. Additionally, no intraoperative or surgical questions were asked; instead, the questions primarily focused on the psychosocial aspects of transplantation. Intraoperative issues, difficulties, and challenges were not mentioned, which would undoubtedly bring about new insights. Future interviews should include questions related to surgical technique, intraoperative complications, and the interplay between various specialties in the OR.

Other important points not addressed in the work are the abilities and skills of the patients. Considering these would certainly provide more insights and a better understanding of what to look for when selecting patients. A study focusing primarily on patients, their characteristics, abilities, and traits would undoubtedly significantly impact the field of VCA.

Furthermore, a detailed discussion of ethical and political aspects was also missing from the interview questions. Future studies should also place greater emphasis on surgical aspects such as intraoperative challenges, team dynamics, as well as pre-, intra-, and postoperative difficulties. Additionally, immersing the patients’ abilities and skills could provide valuable insights and enhance understanding of VCA outcomes Addressing these aspects can enhance both pre- and post-transplantation processes and treatment plans.

## Conclusions

Team science plays a critical role in establishing and maintaining a VCA program. Complex vertical and horizontal communication webs between teams and important. Interdisciplinary cooperation, team-building, and shared decision-making are all important components to success.

## Data Availability

The raw data supporting the conclusions of this article will be made available by the authors, without undue reservation.
